# Telehealth Services Among Active Component Members of the U.S. Armed Forces, 2020–2024

**Published:** 2025-09-20

**Authors:** Adewumi Adegboye, Sithembile L. Mabila

**Affiliations:** Defense Health Agency, Epidemiology and Analysis Branch, Armed Forces Health Surveillance Division, Public Health Directorate, Defense Health Agency, Silver Spring, MD: Mr. Adegboye, Dr. Mabila

Telehealth in the Military Health System (MHS) has long been an important tool for providing care in deployed and non-deployed settings. ^1^ The U.S. Department of Defense uses telehealth for primary care, medication management, ^2^ and other services including out-patient care. Certain types of care provided at fixed military hospitals and clinics, as well as health care encounters outside the military medical system that are billed to TRICARE, are also provided through telehealth. ^3^

This Surveillance Snapshot presents trends in telehealth service use and identifies the 10 most frequent diagnoses addressed via telehealth among U.S. active component service members (ACSMs) using Defense Medical Surveillance System (DMSS) outpatient and demographic records from January 2020 through December 2024.

Telehealth services were identified by having a virtual appointment type or by using the Common Procedural Terminology (CPT) code modifiers 98966–98969, 99374–99380, 99339–99444, 99421–99423, 98000–98007, G0320–G0321, G0425–G0427, G0459, G0508–G0509, D9995, G2061–G2063, C7900–C7902, T1014. The use of telehealth was defined as having at least 1 telehealth encounter per patient per day; if a patient had multiple telehealth encounters per day, the first record was retained as the qualifying encounter. Reasons for telehealth encounters among ACSMs were determined using International Classification of Diseases, 10th Revision codes associated with each telehealth visit. The rate of telehealth encounters was calculated per 10,000 encounter records and stratified by year, patient demographics, and type of care (military clinics or purchased care).

A total of 2,924,428 telehealth encounters were provided to over 1,007,453 ACSMs during the study period. The overall crude rate of telehealth per 10,000 encounters demonstrates an upward trajectory from 2020 to 2024, rising from 228.3 to 515.3 **(Table)** . Over the 5-year study period, women used telehealth at a higher rate than men (345.0 vs. 304.7 per 10,000 encounters, respectively). Direct care from military hospitals and clinics accounted for most telehealth encounters from 2020 through 2024.

**Table:** Rate of Telehealth Services ^a^ by Year, Active Component U.S. Service Members, 2020–2024

Variable	2020	2021	2022	2023	2024	Total
Overall rate ^a^	228.3	223.5	277.5	367.6	515.3	315.3
Sex						
Male	207.8	203.5	266.6	364.9	523.7	304.7
Female	286.9	281.7	307.5	374.9	492.7	345.0
Age group, y						
<20	128.9	109.0	163.9	226.3	358.1	180.3
20–24	233.1	232.2	288.0	374.8	516.3	315.5
25–29	252.7	245.1	296.6	391.8	552.3	340.8
30–34	248.2	241.0	299.3	400.0	560.2	346.2
35–39	236.9	230.3	282.4	375.7	525.1	328.0
40+	203.0	198.8	242.8	324.6	456.9	283.9
Race and ethnicity						
White, non-Hispanic	227.5	219.7	276.2	371.4	525.7	314.0
Black, non-Hispanic	239.6	229.4	261.4	338.3	475.0	302.2
Hispanic	223.7	225.7	285.1	366.6	508.8	319.9
Other	220.1	227.8	296.8	397.6	543.8	334.6
Servicen branch						
Army	176.6	165.7	202.0	248.6	365.8	224.9
Navy	90.3	122.5	176.8	314.1	415.6	221.7
Air Force	511.2	464.5	487.5	578.5	862.6	571.5
Marine Corps	82.3	132.4	244.6	381.9	475.9	248.8
Coast Guard	54.8	137.7	384.2	408.8	421.6	301.2
Space Force ^b^	—	—	610.6	967.3	1185.8	1060.3
Source of care						
Military clinic	279.5	279.4	371.2	535.1	771.2	422.3
Purchased care	18.8	13.8	11.7	10.3	8.9	12.2

a Crude rates were calculated per 10,000 medical encounters.

b Space Force data are available beginning 2022.

The leading 10 reasons for telehealth encounters from 2020 through 2024 were other general symptoms and signs, encounter for other administrative examinations, encounter for immunization, occupational Health Periodic Health Assessment examination, obstructive sleep apnea, low back pain, other specified counseling, pain in right knee, adjustment disorder with mixed anxiety and depressed mood, and pain in left knee (data not shown).

The highest rates observed in 2024 were among male ACSMs (523.7 per 10,000 encounters), those aged 30-34 years (560.2 per 10,000 encounters), Space Force ACSMs (1,185.8 per 10,000 encounters), those treated in a military clinic (771.2 per 10,000 encounters), and ACSMs of other races or ethnicities (543.8) **(Table)** .

The steady increase of telehealth encounter rates from 2020 through 2024 indicates a growing role for virtual care among ACSMs.
